# Case report: targeted whole exome sequencing enables the first prenatal diagnosis of the lethal skeletal dysplasia Osteocraniostenosis

**DOI:** 10.1186/s12881-019-0939-z

**Published:** 2020-01-07

**Authors:** Lara Pemberton, Robert Barker, Anna Cockell, Vijaya Ramachandran, Andrea Haworth, Tessa Homfray

**Affiliations:** 1 Foundation Programme, University College London Hospital, 235 Euston Road, London, UK; 2 Department of Radiology, Frimley Park Hospital, Camberly, UK; 3 Department of Obstetrics, Frimley Park Hospital, Camberly, UK; 4 Congenica Genome Based Medicine, St George’s University Hospital, London, UK; 5 Department of Genetics, St George’s University Hospital, London, UK

**Keywords:** Targeted exome sequencing, Osteocraniostenosis, Prenatal diagnosis

## Abstract

**Background:**

Osteocraniostenosis (OCS) is a rare genetic disorder characterised by premature closure of cranial sutures, gracile bones and perinatal lethality. Previously, diagnosis has only been possible postnatally on clinical and radiological features. This study describes the first prenatal diagnosis of OCS.

**Case presentation:**

In this case prenatal ultrasound images were suggestive of a serious but non-lethal skeletal dysplasia. Due to the uncertain prognosis the parents were offered Whole Exome Sequencing (WES), which identified a specific gene mutation in the FAMIIIa gene. This mutation had previously been detected in two cases and was lethal in both perinatally. This established the diagnosis, a clear prognosis and allowed informed parental choice regarding ongoing pregnancy management.

**Conclusions:**

This case report supports the use of targeted WES prenatally to confirm the underlying cause and prognosis of sonographically suspected abnormalities.

## Background

Osteocraniostenosis (OCS) was first described in 1994 and was noted to be a perinatally lethal condition with premature closure of cranial sutures and gracile bones [1]. OCS is allelic to the less severe Kenny Caffey syndrome (KCS), which is also characterised by impaired skeletal development with small, thin skeletal bones, increased bone density and hypocalcaemia. KCS and OCS are caused by heterozygous mutations in the FAMIIIA gene [2].

Previously, OCS has only been diagnosed postnatally with a combination of typical dysmorphic features, presence of hypocalcaemia and radiological features [3]. Here, we describe the first prenatal diagnosis of OCS.

## Case presentation

**Fig. 1:**
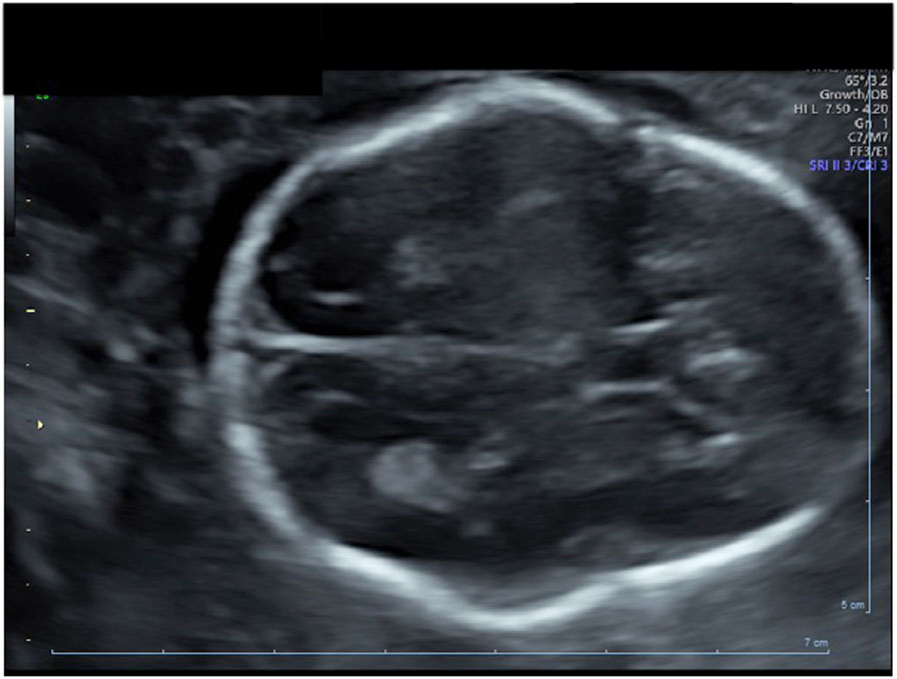
Ultrasound images demonstrate abnormal skull shape with open cranial sutures at 20 weeks gestation

A non-consanguineous couple, who both had healthy children with previous partners, presented during their first pregnancy. The first trimester scan was normal and combined screening showed low risk for a major chromosomal abnormality. The 20-week ultrasound scan identified shortened long bones and an unusual skull shape (Fig. 1). Following a normal detailed chromosome analysis by Comparative Genomic Hybridisation, the couple were referred to clinical genetics.

A cloverleaf skull is often associated with OCS. A subsequent ultrasound scan confirmed an unusual skull shape; however the features were not consistent with a typical cloverleaf skull. All the long bones were shown to be 5 Standard deviations (SD) below the mean and the head and abdominal circumference were 2 SD below the mean. The ultrasound images did not support a diagnosis of Diastrophic Dysplasia. While the images suggested possible curving of both radii and bilateral Hitchhiker’s thumb, ear morphology was normal and no talipes (club foot) was identified. The findings were suggestive of a skeletal dysplasia without a narrow chest or fetal hydrops, therefore a lethal skeletal dysplasia was not initially suspected. The couple were counselled about the uncertainty of the child’s prognosis and offered targeted whole exome sequencing (WES).

Trio exome sequencing was performed with parental DNA and the stored fetal DNA extracted from the amniotic fluid at 21 weeks. Targeted WES was performed using the Agilent Clinical Research Exome Version 2 & sequencing on the Illumina NextSeq500. This took 15 days and identified a de novo mutation in FAMIIIA. The mutation identified, c.1026_1028delTTC p.(ser343del), has previously been identified in two unrelated male patients with Osteocraniostenosis [ 4]. In both cases, the mutation was lethal within the first month of life and associated with thin dense bones with poor bone marrow formation.

**Fig. 2:**
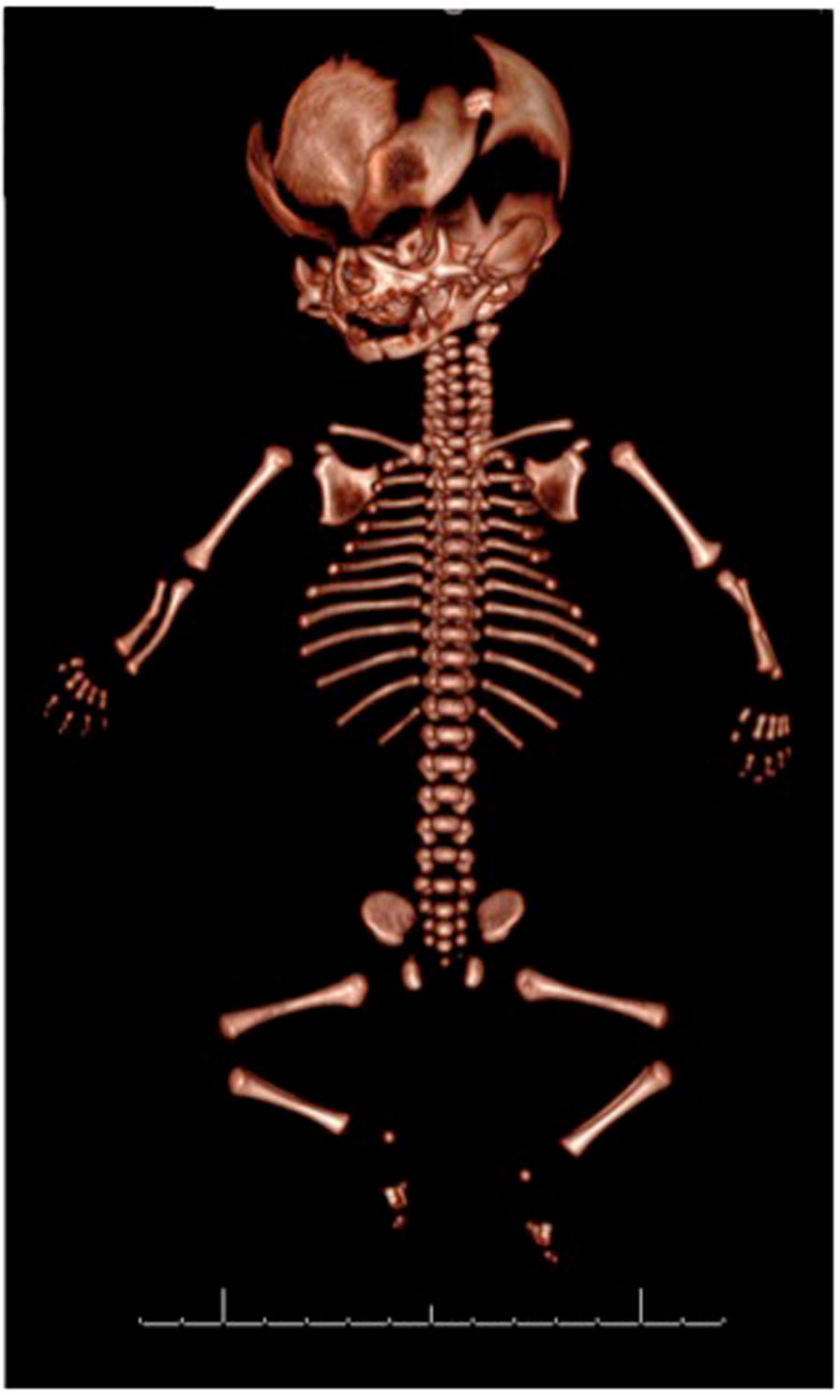
Post delivery CT images confirming short thin gracile bones and the abnormal skull with basal cranialstenosis

Targeted WES accurately diagnosed a lethal skeletal dysplasia that had not been suspected from the ultrasound features. This provided essential prognostic information and allowed parental choice regarding further pregnancy management. Following the lethal skeletal dysplasia diagnosis, the couple made the difficult decision to end the pregnancy. The postnatal 3D CT scan confirmed the skeletal abnormalities that were detected in the prenatal ultrasound. (Fig. 2).

## Discussion and conclusion

Prenatal diagnosis helps guide parents and healthcare professionals regarding pregnancy management decisions, delivery options and postnatal treatment [5].The introduction of next generation sequencing has exponentially changed the ability to definitively diagnose monogenic diseases caused by de novo and recessive mutations. Approximately 1–2% of the human genome encodes the exome but it is thought to contain more than 85% of all disease causing mutations [5].

Currently there is not an identifiable syndrome associated with mutations in all genes. Therefore, in the clinical setting, targeted WES is used. This limits analysis to those genes that have mutations compatible with the phenotype identified on ultrasound. Prenatal WES relies on this sonographic evidence to help select the most appropriate Human Phenotypic Ontology (HPO) terms in order to target the analysis appropriately. Despite advances in ultrasound, accurate prenatal diagnosis is limited to those syndromes with a characteristic phenotype such as thanatophoric dysplasia and achondroplasia.

Whole Exome Sequencing (WES) has frequently been used postnatally to diagnose rare syndromes however experience with prenatal WES remains limited. Analysis and interpretation of the exome data has been too slow to be helpful in the prenatal setting, which requires rapid results. Recent publications such as the PAGE study 2019 and Petroski et al. 2019 have confirmed the diagnostic potential of prenatal exome sequencing [6, 7]. The majority of cases reported thus far have only reported the results after the pregnancy [8]. The introduction of rapid targeted exome sequencing with results available within 3 weeks allows for prenatal management.

One of the concerns raised by the use of WES is the potential for secondary findings. These are genetic variants that are not related to the phenotype in question, such as finding cancer susceptibility gene mutations when looking for genes associated with skeletal dysplasia. Interpretation of secondary findings are not always clear and can lead to ethical dilemmas in further counselling [9] . The use of targeted WES using phenotypic gene panels not only significantly reduces incidental/ secondary findings but also accelerates data interpretation [10]. In this case, no secondary findings were identified as only genes associated with skeletal abnormalities were examined. It was explicit in the consent that we would not find or report incidental findings.

WES is a powerful tool for accurate prenatal diagnosis, but it must be done after the 2nd trimester ultrasound. If a 2nd trimester ultrasound is performed at 20–22 weeks gestation and the WES results take 3 weeks, then the prenatal choices may be limited for couples who live in countries with more stringent pregnancy termination laws. For WES to be used successfully, good phenotyping is essential. This requires close co-operation between genetics and fetal medicine.

The diagnosis of OCS is currently made in the postnatal period by gross exam and imaging. We used targeted WES to achieve the first reported case of a prenatal diagnosis of OCS. This demonstrates that, when the prognosis of a fetus is uncertain, the prenatal targeted WES could be used as a tool to help with management and treatment decisions.

## Data Availability

The datasets analysed during the current study are available in the Decipher repository. https://decipher.sanger.ac.uk/browser#q/FAM111A/location/11:58791366-59041366

## Abbreviations

HPO: Human Phenotypic Ontology
KCS: Kenney Caffey Syndrome
OCS: Osteocraniostenosis
SD: Standard Deviations
WES: Whole Exome Sequencing
